# IgA immunoglobulin isotype of rheumatoid factor in primary Sjögren’s syndrome

**DOI:** 10.1007/s00296-020-04782-3

**Published:** 2021-01-26

**Authors:** Maria Maślińska, Małgorzata Mańczak, Brygida Kwiatkowska, Vince Ramsperger, Long Shen, Lakshmanan Suresh

**Affiliations:** 1 National Institute of Geriatrics, Rheumatology and Rehabilitation, Warsaw, Poland; 2 Department of Gerontology and Public Health, National Institute of Geriatrics, Rheumatology and Rehabilitation, Warsaw, Poland; 3 Department of Oral Diagnostic Sciences, State University of New York at Buffalo, Buffalo, NY USA; 4 Trinity Biotech Plc., Buffalo, NY USA

**Keywords:** Rheumatoid factor, Isotype IgA, Primary Sjögren’s syndrome

## Abstract

Primary Sjögren’s syndrome (pSS) is an autoimmune disease with autoantibodies overproduction, including rheumatoid factors (RF). RF-IgA, IgG immunoglobulin classes are suggested as potential biomarkers of pSS. We studied 76 patients with pSS (ACR/Eular 2017); laboratory tests included ESR, C-reactive protein, concentrations of gamma globulins, RF, Anti-SS-A/Ro, and anti-SS-B/La. Eye dryness and keratoconjunctivitis sicca were confirmed with Schirmer’s test, the ocular staining score (OSS) using lissamine green, fluorescein staining and biopsy of minor salivary gland with the histopathological evaluation. Differences between groups were analyzed with *U* Mann–Whitney test. Correlations between quantitative variables were assessed with the Spearman correlation coefficient.. The best diagnostic values of immunoglobulin concentration for discriminating pSS patients and healthy individuals are for RF-IgA. With cut-off of 21.5 EU/mL, the sensitivity is 72% and specificity is 100%. Very high specificity (100%) is also obtained for RF-IgM concentration of 74.1 EU/mL. Sensitivity is, however, smaller than that for RF-IgA and amounted to 61%. The RF-IgG is the poorest indicator of pSS with 51% of sensitivity and 95% of specificity. To summarize RF-IgA strongly associate with anti-SS-A and anti-SS-B autoantibodies. Both RF-IgA and RF-IgM may be used as diagnostic tools for pSS. Conclusions: among the three studied rheumatoid factor subtypes, RF-IgA showed the best diagnostic accuracy for pSS. RF-IgA correlated with anti-SS-A/Ro and anti-SS-B antibodies even more closely than RF-IgM. The assessment of the RF-IgA serum concentration may be helpful in the process of establishing pSS diagnosis.

## Introduction

Primary Sjögren’s syndrome (pSS) is an autoimmune disease characterized by external exocrine glands dysfunction, multiorgan involvement and production of autoantibodies including, among others, rheumatoid factors (RF). Rheumatoid factors are the immunoglobulins of various isotypes (IgM, IgG, IgA) with their activity directed against antigenic sites in the Fc region of human IgG. The main isotype of rheumatoid factor is IgM (RF-IgM), the presence of which is clinically determined using available diagnostic tests for RF detection [1]. RF-IgM is found in about 80% of patients with rheumatoid arthritis (RA) but is also present in about 1–4% of the general population. The incidence of RF increases with age in the general population [1, 2]. The presence of RF may accompany an immune system response to infectious and non-infectious factors, such as mononucleosis, tuberculosis, syphilis, leprosy, malaria, leptospirosis or sarcoidosis [3, 4]. The prevalence of RF-IgM varies in autoimmune inflammatory rheumatic diseases (AIRD) other than RA, but in patients with pSS it is particularly common (70–90%) and may be present in high concentration in patients’ sera [1, 2].

Rheumatoid factor IgM has established meaning as well as having diagnostic and prognostic uses for RA [5, 6]. Rheumatoid factor of other than IgM immunoglobulin classes, such as IgA or IgG, have been proposed as a prognostic factor of a more severe course of RA with faster progression of radiological changes (erosions, destruction)[5]. It was even suggested, that finding RF IgA in the initial stage of RA because of its prognostic significance should result in the introduction of more aggressive methods of RA treatment [6]. In addition, elevated RF IgG levels in sera of RA patients has been associated with arthritis and vasculitis [7].

The simultaneous presence in patient’s serum of both IgM and IgA RF isotypes and IgG anti-CCP2 antibodies was proposed to have a 100% Positive Predictive Value (PPV) for RA diagnosis [7].

In pSS, the association of RF IgM with disease activity has been already described. RF IgA and IgG immunoglobulin classes have been suggested as potential biomarkers of immunological and clinical pSS features [8–10]. It was reported that RF-IgA is more often present in pSS, than in RA [11].

### Objectives

The purpose of the present study was to examine correlations between RF-IgA concentrations in sera of pSS patients and the levels of other autoantibodies (including RF IgM and IgG isotypes) with the results of dry eye tests, histopathological assessment of stages of inflammation in minor salivary gland lip biopsy (focus score, FS) and basic laboratory tests results.

## Materials and methods

We studied 76 patients, mean age 53 (SD = 13); (*n* = 67; 88% Female/*n* = 9; 12% Male, female/male ratio 7.4:1) with pSS diagnosis (criteria ACR/Eular 2017); mean disease duration from diagnosis 2.24 years. The control group consisted of 19 healthy volunteers, recruited from among hospital employees, with mean age 43.5 (SD = 16); (n16; 84%/Female/*n* = 4; 16% Male).

Patients with other rheumatic diseases were excluded from the analysis as a control group, because of a possibility of the secondary SS development, which would influence the clarity of the analysis and create a risk of the overlap syndromes e.g. pSS with RA or LE.

Osteoarthritis (OA) was not taken into account, as this disease is a process without systemic symptoms which does not affect general inflammation and serological tests. The mean age of patients (57 year old) and also the mean age of a control group (43 year old) may indicate, that among studied subjects in both groups many were suffering from asymptomatic OA.

Laboratory tests performed: erythrocyte sedimentation rate (ESR), C-reactive protein (CRP) concentration (mg/L), concentrations of gamma globulins (g/dL), white blood cell count (WBC), alanine aminotransferase (ALAT) (U/L) and asparagine aminotransferase (AspAT) serum activity U/L, presence of cryoglobulins, serum concentration of C3, C4 components of complement (mg/dL), pH and urine specific gravity. Rheumatoid factor serum concentration was assessed using ImmuLisa Enhanced™ RF IgA, IgG, IgM antibody ELISA (IMMCO Diagnostics, Inc., Buffalo, NY, USA). The positive result for the RF-IgA and RF-IgG concentration was > 25 EU/mL (with borderline between 20 and 25 EU/mL and highly positive result set as ≥ 100 EU/mL). The positive result for RF-IgM was > 12.5 EU/mL and highly positive ≥ 50 EU/mL. Antinuclear antibodies (ANA) were detected using indirect immunofluorescence on HEp-2 cells for cytoplasmic pattern; no additional titration was performed after the initial 1:40 screen. Anti-Ro/SS-A, and anti-La/SS-B antibodies were detected by quantitative ELISA (IMMCO Diagnostics, Inc., Buffalo, NY) tests with reference range for anti-SS-A/Ro antibodies: negative: < 20 EU/mL; borderline: 20–25 EU/mL; positive: > 25 EU/mL; and for anti-SS-B/La-antibodies: negative: < 50 EU/mL; positive: ≥ 50 EU/mL. The eye dryness and keratoconjunctivitis sicca were confirmed with Schirmer’s test (positive-score of less than 5 mm/5′) and the ocular staining score (OSS) using lissamine green and fluorescein staining. Lower lip biopsy of minor salivary glands with the histopathological evaluation and infiltration by 50 or more mononuclear cells on 4 mm^2^ biopsy surface was considered as focus score (FS) 1.

### Ethical standards

The study was approved by the National Institute of Geriatrics, Rheumatology and Rehabilitation Bioethics Committee, approval from 1 December 2016. The informed consent from all studied subjects was obtained.

### Statistical analyses

The normality of the distribution of continuous variables was verified using the Shapiro–Wilk test. Data are expressed as median and interquartile range (IQR) because of the skewness of distributions. Mann–Whitney test was used to assess differences between pSS patients and control group. Correlations between variables were calculated using the Spearman correlation coefficient (*ρ*). An analysis of the ROC (receiver operating characteristic) curve has been carried out to determine ability of RF IgA, IgG, IgM values to classify a person in the pSS group. Optimal cut-off points were chosen guided by the Youden index maximum and high value of specificity. Sensitivity, specificity, positive predictive value (PPV) and negative predictive value (NPV) were calculated. Positive predictive value (PPV) and negative predictive value (NPV) were calculated for 95% confidence interval (95%CI).

Data were analyzed using Statistica v13.1 Dell Inc. 2016.

## Results

Immunoglobulin serum concentrations were higher in pSS group than in the control group. Table 1 presents significant differences (*p* < 0.05) observed between both analyzed groups in concentrations of particular immunoglobulin subclasses of RF.

**Table 1:** Comparison between pSS patient group and healthy controls in term of RF-IgA, RF-IgG and RF-IgM concentrations

Concentration	pSS *n* = 76 median (IQR)	Control group *n* = 19 median (IQR)	*p* value
RF IgA—EU/mL	102.27 (18.41–275.30)	6.17 (4.71–9.57)	< 0.001
RF IgG—EU/mL	26.68 (16.90–49.49)	16.85 (13.65–21.07)	0.001
RF IgM—EU/mL	112.22 (20.46–209.58)	13.99 (5.68–22.47)	< 0.001

Analysis showed that none of the healthy subjects had positive RF-IgA and only 11% had positive RF-IgG; 59% had positive RF-IgM. In comparison 66% of pSS group had positive RF-IgA, 55% RF-IgG and 88% RF-IgM. Table. 2

**Table 2:** Summarized analysis of RF concentrations in positive values (according to laboratory reference range) between studied groups

Concentration	pSS *n* = 76	Control group *n* = 19	*p* value
RF IgA—EU/mL (positive > 25)	50 (66%)	0 (0%)	< 0.001
RF IgG—EU/mL (positive > 25)	42 (55%)	2 (11%)	0.001
RF IgM—EU/mL (positive > 12.5)	67 (88%)	11 (59%)	0.005

The highly positive (++) concentration of RF-IgA was found in 39 pSS patients, which constituted a 51% of a whole pSS group and 78% of all RF-IgA positive patients. The highly positive RF-IgG was found in 9% (*n* = 7) of pSS patients. Over 62% (*n* = 47) of pSS group had high concentration of RF-IgM. There were no significant differences between patients with RF-IgA positive (+) and RF-IgA negative (−), that would concern any of the non-serologic variables that are included in the pSS classification criteria, that is: FS, OSS and Schirmer’s test. There were significantly higher anti—SS-A/Ro antibodies concentrations (*p* = 0.002) in RF-IgA positive group. The difference in anti—SS-A/Ro antibodies level (*p* = 0.028) was even more significant, when we compared RF-IgA(−) with RF-IgA(++). Comparing groups RF-IgM(+) vs. RF-IgM(−) we found, that in patients with RF-IgM (+) the concentration of anti -SS-A/Ro antibodies was statistically higher (*p* = 0.007). Analysis of RF-IgG (+) vs. RF-IgG(−) showed, that RF-IgG(+) subgroup revealed significantly higher concentration of anti-SS-B/La antibodies (*p* = 0.015). Positive correlation was found between concentrations of: RF-IgA and RF-IgG (*ρ* = 0.647) and RF-IgA and RF-IgM (*ρ* = 0.669). Correlations of RF-IgA serum concentrations with: ANA antibodies, concentrations of anti-SS-A/Ro, anti-SS-B/La antibodies, other studied RFs, high serum concentrations of gamma globulins (> 1.5 g/dL) and ESR > 20 mm/h—are presented in Table 3.

**Table 3:** RF-IgA correlations with serum level of this autoantibodies and anti-SS-A/Ro, anti-SS-B/La antibodies, other studied RFs, high serum gamma globulin concentrations (> 1.5 g/dL) and ESR > 20 mm/h

	ln ANA	Anti-SS-A/Ro	Anti-SS-B/La	RF-IgG EU/mL	RF-IgM EU/mL	γglobulins > 1.5 g/dL *n* = 32	ESR > 20 mm/h *n* = 37
RF-IgA EU/mL	*ρ* = 0.295 *p* < 0.05	*ρ* = 0.461 *p* < 0.01	*ρ* = 0.500 *p* < 0.01	*ρ* = 0.647 *p* < 0.01	*ρ* = 0.669 *p* < 0.01	*ρ* = 0.178 *p* > 0.05	*ρ* = 0.290 *p* > 0.05

*AUC* area under the curve, *PPV* positive predictive value, *NPV* negative predictive value, *RF* rheumatoid factor

**Fig. 1:**
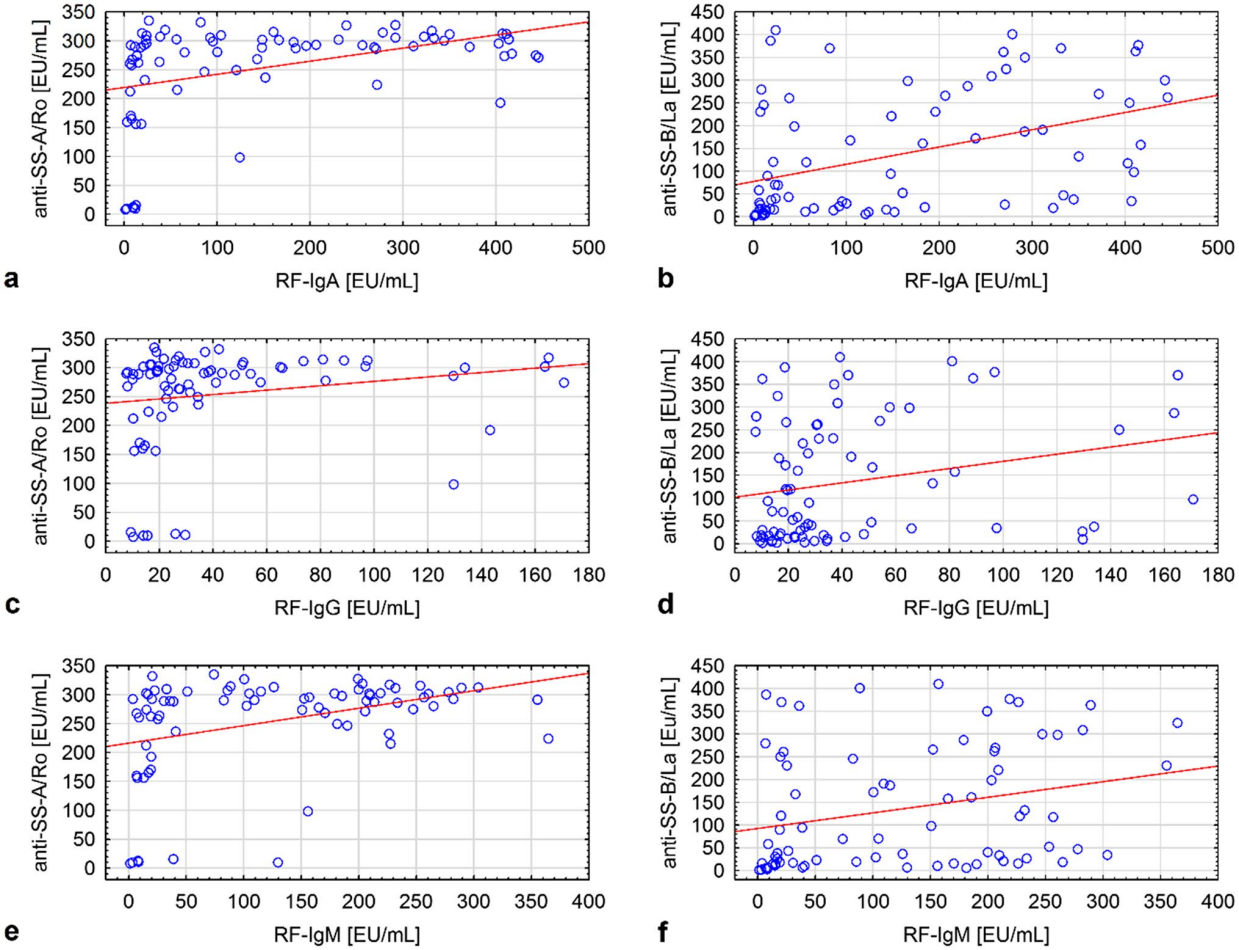
Scatter plots of RFs in three immunoglobulin classes against anti-SS-A/Ro and anti-SS-B/La antibodies

There were positive correlation between concentrations of: RF-IgM and RF-IgG and anti-SS-A/Ro, anti SS-B/La antibodies (*ρ* = 0.390 and *ρ* = 0.331, respectively, for anti SS-A/Ro; *ρ* = 0.344 and *ρ*  = 0. 324, respectively, for anti-SS-B antibodies). Figure 1a, b, c, d, e, f presents scatter plots of RFs in three immunoglobulin classes against anti-SS-A/Ro and anti-SS-B/La antibodies.

Of RF-IgM positive patients (*n* = 67), 50 (75%) had RF-IgA. However, when analyzing the group of RF-IgA positive patients (*n* = 50), it was found that all of them had also RF-IgM (100%).

The 88.2% (*n* = 67) of pSS patients had positive ANA–ANA titer 1: 1280 had 68% (*n* = 52) (IQR: 1:640–1:1280). Up to 92% (*n* = 70) of pSS patients had anti-SS-A/Ro antibodies, while anti-SS-B/La were present in 55.3% (*n* = 42) of pSS group.

In the control group ANA positivity was observed only in 5 subjects with low titer (1:40) and cytoplasmic (*n* = 4) and speckled (*n* = 1) pattern. None of studied healthy subjects had anti-SS-B/La antibodies and only four among of individuals presenting low ANA positivity had anti-SS-A antibodies detected. There were no significant correlation of studied variables with RFs in the control group.

**Fig. 2:**
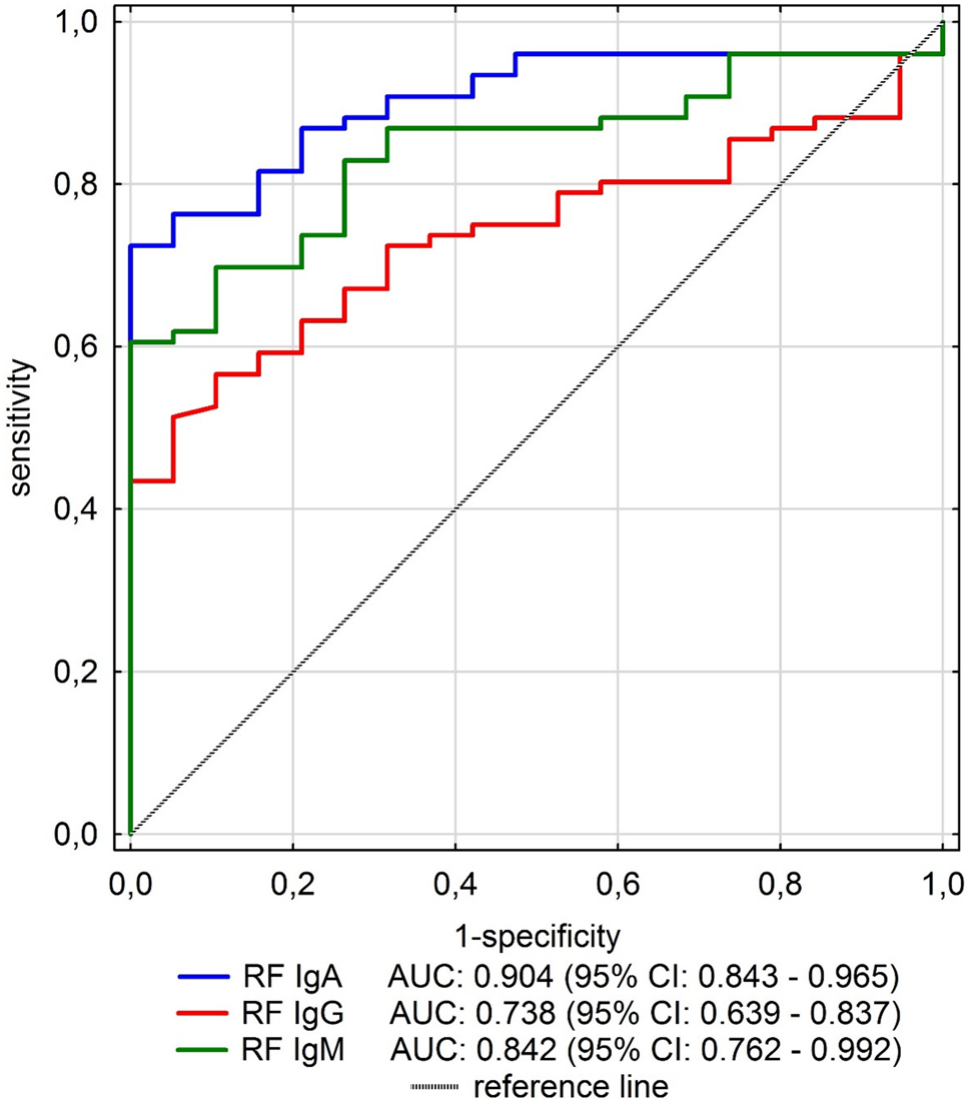
Receiver operating characteristic (ROC) curves for RF-IgA, RF-Ig-G and RF-IgM

There were no correlations found in pSS group between concentrations of RF-IgA, RF-IgG with patient’s age and laboratory results such as: WBC, C4, C3 complement components, ALAT, AspAT, presence of cryoglobulins, pH and urine specific gravity or results of FS, OSS, and Schirmer’s test. Receiver operating characteristic (ROC) analysis for RF-IgA, RF-Ig-G and RF-IgM as predictors of pSS were performed. The largest area under the curve (AUC) was for RF IgA: 0.904 (95% CI 0.843–0.965) which was shown on Fig. 2.

Diagnostic characteristics of RFs concentrations for pSS prediction are presented in Table 4. There are two cut-offs values for each RF: the first one selected with Youden index, the second one is the value from our data, which is the nearest to the norm limit of particular RF.

**Table 4:** Sensitivity, specificity, and predictive values of RFs as indicators of pSS

	Cut-off	Sensitivity (95% CI)	Specificity (95% CI)	PPV (95% CI)	NPV (95% CI)	Youden index
RF-IgA AUC = 0.904	21.5	72 (60–82)	100 (82–100)	100	48 (39–57)	0.724
	24.1	67 (55–77)	100 (82–100)	100	43 (36–51)	0.671
RF-IgG AUC = 0.738	26.1	51 (40–63)	95 (74–100)	98 (85–100)	33 (27–39)	0.461
	25.1	55 (43–67)	90 (67–99)	96 (85–99)	33 (27–40)	0.447
RF-IgM AUC = 0.842	74.1	61 (49–72)	100 (82–100)	100	39 (32–46)	0.605
	13.2	88 (79–94)	42 (20–66)	86 (80–90)	47 (28–67)	0.303

The best diagnostic values of immunoglobulin concentration for discriminating pSS patients and healthy individuals are for RF-IgA. With cut-off of 21.5 EU/mL the sensitivity is 72% and specificity 100%. Very high specificity (100%) is also obtained for RF-IgM concentration of 74.1 EU/mL. Sensitivity is, however, smaller than for RF-IgA and amounted to 61%. The RF-IgG is the poorest indicator of pSS with 51% of sensitivity and 95% of specificity.

## Discussion

Primary Sjogren’s syndrome is an autoimmune disease with strong predilection for RF presence, i.e. RF-IgM [12, 13] and presently RF remains an important element of prognosis and a maker of immunological activity in pSS.

In our previous study, focused on classical RF (RF-IgM), we described its correlation with more severe course of pSS (lower Schirmer’s test, higher ESSDAI, leucopenia, higher level of gammaglobulins, ANA titer, anti-SSA and anti-SS-B autoantibodies) [13]. The studies by other authors supported our findings [14]. Results of some studies suggested, that RF-IgA may indicate more severe bone involvement/inflammation and the extraarticular manifestations of this autoimmune disease [14, 15]. Another study revealed, that the assessment of anti-citrulinated peptide antibodies (ACPA) and the RF-IgA together with IgM-RF had no advantage, in regard to the specifity and senitivity in diagnosing RA, than the assesment of RF-IgM only [16].

Patients with pSS were also widely analyzed for RF in other than IgM classes of immunoglobulins. In the work by Peen E et al. [17] out of 97 studied pSS patients 25.8% were RF-IgA positive and among them 80% were highly positive. In our work up to 66% of the whole pSS patients group (*n* = 76) had RF-IgA out of which 78% had highly positive results (which amounts to 51% of whole pSS group). What seems to be particularly important, is that in our control group none of the subjects had RF-IgA, while in control group in the Peen et all study only 1% (*n* = 1 out of 100).

In the presented study RF-IgA concentrations closely correlated with pSS marker autoantibodies e.g. anti-SS-A antibodies had higher correlation with RF-IgA than with RF-IgM (*ρ* = 0.461 and *ρ* = 0.390 respectively). Interestingly all pSS patients with anti RF-IgA also had RF-IgM, indicating that RF-IgA will not be useful in the pSS diagnosis of RF-IgM negative patients.

In 1989 Muller et al. [18] analyzed 40 patients with pSS describing the prevalence of RF-IgA similar to that found in our study. In the study by Muller et al. as much as 55% of patients had highly positive results of RF-IgA concentrations, with all those individuals testing positive for RF-IgM as well. In the same study RF-IgA correlated strongly positively with anti-SS-A and anti -SS-B antibodies, although the correlation of those antibodies with RF-IgM was shown to be stronger. The authors also presented the positive correlation of RF-IgA with hypergammaglobulinemia. In our work such a relationship was not confirmed. Interesting work was presented by Lee et al. [19] performed on 77 pSS patients diagnosed according to ACR/EULAR(2016) pSS classification criteria and a control group of subjects with idiopathic sicca syndrome. Interestingly, the authors used the same ELISA tests for determination of RF-IgM and RF-IgA serum concentration. This study confirmed high prevalence for RF-IgA presence in pSS, but also revealed RF-IgA in 21% of patients from a sicca group. The same percentage of pSS patients tested positive for RF-IgA as for IgM-RF—which is a result similar to our current study. RF-IgM was present in as much as 86.5% of patients with dryness. The sicca group from Lee et al. study, compared to a pSS group, was shown to be older, tested negative for autoantibodies (ANA, anti-SS-a, anti-SS-B, concentration of complement components, RF-IgA and RF-IgG) and had significantly lower RF-IgM concentration levels. This group may therefore differ from the general population in terms of age—being older, showing more frequent RF-IgM presence but at low concentrations. These authors showed the ROC curve of the diagnostic accuracy for serum RF-IgA concentration with AUC 0.867 (95% CI 0.795, 0.938) [19]—which is similar to our work which revealed RF-IgA concentration: 0.904 (95% CI 0.843–0.965). In the cited article—as well as in our study—there was no association of RF-IgA with age, disease duration or xerophthalmia. In both current and Lee et al. [19] studies the correlation of RF-IgA with immunological disease activity (ANA, anti-SS-A, anti-SS-B antibodies, RF-IgG and Ig-M) was confirmed. We didn’t confirm a correlation of RF-IgA with C3 component of complement level, which was shown in the study by Lee et al. Both studies show no correlation between RF-IgA and WBC, ESR, urine assessment and xerophthalmia. It is worth noticing, that a relatively high sensitivity and specificity of RF-IgA results determined by Youden’s index indicate, that results falling within a borderline range between 20 and 25 EU/mL should be considered as positive.

As the destruction of the mucosal barrier underlies pSS pathogenesis, the involvement of IgA in the process a so-called mucosal immunoglobulin, also present in exocrine secretions such as tears or saliva came under scrutiny. However, results of performed studies for the presence of RF-IgA in tears or saliva in pSS were contradictory.

Markusse et al. [20] showed the increased level of RF-IgA (but not RF-IgM and IgG) in saliva of pSS patients and suggested its local production. Hung et al. [21] studied the saliva of pSS patients in terms of RF-IgA, Interleukin-6 (IL-6), Interleukin-17A (IL-17A) and tumor necrosis factor (TNF) concentrations, with significant results confirmed only for IL-6.

There are a few studies on the presence of RF-IgA in tears, one of which concerned patients with RA. Its results did not show a correlation between the presence of RF-IgA and the development of secondary Sjogren’s syndrome [22].

Interesting results were presented by Meek et al. [11] who studied RF isotypes and SS-A in both pSS and RA patients with keratoconjunctivitis sicca. The study showed, that in a pSS group IgA-RF concentrations were higher than IgM-RF.

In our research we found no correlation between RF-IgA and eye dryness assessment (OSS, Schirmer’s test) or results concerning the stage of salivary glands inflammation described as FS.

However, it is also necessary to indicate limitation of the presented study resulting from a relatively small control group of healthy individuals, which accounts for 25% of the number of the pSS study group (*n* = 19–*n* = 76).

## Conclusions

Among three studied rheumatoid factor subtypes studied, RF-IgA showed the best diagnostic accuracy for pSS and correlated with anti-SS-A/Ro and anti-SS-B antibodies even more closely than RF-IgM.

As RF-IgA was notably absent in the control group, the research into prevalence of RF-IgA in healthy population is worth considering proving a value of RF-IgA presence as a marker of pathological process.

The present study didn’t confirm the supposed association of RF-IgA in pSS with main features of this disease, such as FS or eye dryness. Still, its presence correlated with level of antibodies, which are characteristic for a serologic profile of pSS: ANAs, anti-SS-A/Ro and anti-SS-B/La. Thus, RF-IgA may be considered as an additional marker of immunological activity in pSS.

Concluding, our research proves, that the assessment of the RF-IgA serum concentration may be helpful in the process of establishing pSS diagnosis.

## Data Availability

The datasets generated during and/or analyzed during the current study are available from the corresponding author on reasonable request.
